# Oncostatin M inhibits differentiation of rat stem Leydig cells in vivo and in vitro

**DOI:** 10.1111/jcmm.13946

**Published:** 2018-10-15

**Authors:** Yiyan Wang, Lubin Xie, Erpo Tian, Xiaoheng Li, Zina Wen, Linchao Li, Lanlan Chen, Ying Zhong, Ren‐Shan Ge

**Affiliations:** ^1^ Department of Anesthesiology The Second Affiliated Hospital and Yuying Children's Hospital Wenzhou Medical University Wenzhou China; ^2^ Jinjiang Maternity and Child Health Hospital Sichuan China

**Keywords:** differentiation, oncostatin M, stem Leydig cells, testosterone

## Abstract

Oncostatin M (OSM) is a pleiotropic cytokine within the interleukin six family of cytokines, which regulate cell growth and differentiation in a wide variety of biological systems. However, its action and underlying mechanisms on stem Leydig cell development are unclear. The objective of the present study was to investigate whether OSM affects the proliferation and differentiation of rat stem Leydig cells. We used a Leydig cell regeneration model in rat testis and a unique seminiferous tubule culture system after ethane dimethane sulfonate (EDS) treatment to assess the ability of OSM in the regulation of proliferation and differentiation of rat stem Leydig cells. Intratesticular injection of OSM (10 and 100 ng/testis) from post‐EDS day 14 to 28 blocked the regeneration of Leydig cells by reducing serum testosterone levels without affecting serum luteinizing hormone and follicle‐stimulating hormone levels. It also decreased the levels of Leydig cell‐specific mRNAs (*Lhcgr*,* Star*,* Cyp11a1*,* Hsd3b1, Cyp17a1* and *Hsd11b1*) and their proteins by the RNA‐Seq and Western blotting analysis. OSM had no effect on the proliferative capacity of Leydig cells in vivo. In the seminiferous tubule culture system, OSM (0.1, 1, 10 and 100 ng/mL) inhibited the differentiation of stem Leydig cells by reducing medium testosterone levels and downregulating the expression of Leydig cell‐specific genes (*Lhcgr*,* Star*,* Cyp11a1*,* Hsd3b1, Cyp17a1* and *Hsd11b1*) and their proteins. OSM‐mediated action was reversed by S3I‐201 (a STAT3 antagonist) or filgotinib (a JAK1 inhibitor). These data suggest that OSM is an inhibitory factor of rat stem Leydig cell development.

## 1 INTRODUCTION

Testicular Leydig cells play a vital role in promoting genital development in the male fetus, maintaining the secondary sexual characteristics, and stimulating the spermatogenesis at adulthood.^1^, ^2^ There are two different populations of Leydig cells in the rodent life‐span: fetal Leydig cells and adult Leydig cells. Fetal Leydig cells develop during the embryonic period and adult Leydig cells differentiate from stem Leydig cells during puberty.^3^, ^4^ The differentiation of adult Leydig cells includes four phases: stem, progenitor, immature and mature stage.^4^ Adult Leydig cells, once formed, rarely divide and slowly maintain its population by spontaneous differentiation from stems Leydig cells.^4^, ^5^ However, when adult Leydig cells are depleted in the rat testis after a single intraperitoneal injection (75 mg/kg) of a drug, ethane dimethane sulfonate (EDS), adult Leydig cells can regenerate rapidly within 2 months.^6^, ^7^ Newly‐formed progenitor Leydig cells arise from stem Leydig cells on the outer surface of the seminiferous tubule.^8^, ^9^ The development of stem Leydig cells into the Leydig cell lineage as the progenitor Leydig cells emerge with the initial up‐regulation of Leydig cell lineage biomarkers: luteinizing hormone receptor (LHCGR, encoded by *Lhcgr*), scavenger receptor class B member 1 (SCARB1, encoded by *Scarb1*), steroidogenic acute regulatory protein (STAR, encoded by *Star*), cytochrome P450 cholesterol side‐chain cleavage enzyme (CYP11A1, encoded by *Cyp11a1*), 3β‐hydroxysteroid dehydrogenase 1 (HSD3B1, encoded by *Hsd3b1*), cytochrome P450 17α‐hydroxysteroid dehydrogenase/17‐20 lyase (CYP17A1, encoded by *Cyp17a1*).^8^ Progenitor Leydig cells further mature into immature Leydig cells with the up‐regulation of 17β‐hydroxysteroid dehydrogenase 3 (HSD17B3, encoded by *Hsd17b3*) and 11β‐hydroxysteroid dehydrogenase 1 (HSD11B1, encoded by *Hsd11b1*).^10^, ^11^, ^12^ The regeneration of Leydig cells was similar to the pubertal developmental process of Leydig cells and it is a unique model for studying the effects of niche factors on Leydig cell development in the adult testis.^11^ Recently, a seminiferous tubule culture system has been established to mimic the in vivo Leydig cell development after the culture of the seminiferous tubules in Leydig cell differentiation medium (LDM) and this system can be used for investigating effects of niche factors on stem Leydig cell differentiation and proliferation.^13^

There are several niche factors that have been identified to regulate the stem Leydig cell development, including platelet‐derived growth factor AA and BB,^13^, ^14^, ^15^, ^16^, ^17^ desert hedgehog,^13^, ^18^, ^19^ insulin‐like growth factor 1 (encoded by *Igf1*),^20^, ^21^ kit ligand,^22^ and cytokines.^23^, ^24^ After initial screening over 40 niche factors, oncostatin M (OSM), a 28‐kDa protein that belongs to a member of the interleukin (IL)‐6 cytokine family, is identified. The IL‐6 family comprises IL‐6, IL‐11, leukemia inhibitory factor (LIF), ciliary neurotrophic factor, cardiotrophin‐1 and neurotrophin‐1/B‐cell stimulatory factor‐3.^25^, ^26^ Their effects are exerted via a common signal transducing receptor chain glycoprotein (gp) 130 (also called IL6ST, encoded by *Il6st*). OSM binds to the type I OSM receptor, which forms a heterodimer with IL6ST to transduce the signal. OSM plays a biological role by activating Janus kinase (JAK)‐signal transduction with transcriptional activator (STAT) signal transduction pathway and mitogen‐activated protein kinase (MAPK) signaling pathway and may be involved in cell growth, differentiation, inflammatory response, hematopoietic processes and tissue remodeling.^27^

OSM is produced mainly by activated T cells, neutrophils, monocytes and macrophages. It has also been found that OSM is highly expressed in the gonads of developing embryos,^28^ in the late fetal and early neonatal rat testis as well as in the maturing and adult testis.^29^ In the rat testis, OSM is mainly present in the Leydig cell lineage and is secreted by immature and adult Leydig cells.^30^ It was found that OSM can regulate Sertoli cell and gonocyte proliferation.^31^, ^32^ Since stem/progenitor Leydig cells are most abundant during the neonatal testis,^8^, ^33^ the role of OSM on stem/progenitor Leydig cell development is unclear. In the present study, we used in vivo EDS‐treated Leydig cell regeneration model and in vitro seminiferous tubule culture system to address the action of OSM and its possible mechanisms.

## 2 MATERIALS AND METHODS

### 2.1 Chemicals and kits

OSM was purchased from PeproTech (Rocky Hill, NJ, USA). Immulite2000 Total Testosterone Kit was purchased from Sinopharm Group Medical Supply Chain Services Co. (Hangzhou, Zhejiang, China). Filgotinib and S3I‐201 were purchased from Selleck (Shanghai, China). The culture media (M‐199 and DMEM/F12) and Click‐iT EdU (EdU) imaging kit were purchased from Invitrogen (Carlsbad, CA, USA). LH was a gift of NIDDK (Bethesda, MD, USA). EDS was purchased from Pterosaur Biotech (Hangzhou, China). All other reagents were obtained from Sigma‐Aldrich (St. Louis, MO, USA).

### 2.2 Animal treatment in vivo

Male Sprague‐Dawley rats (63 days of age) were purchased from Shanghai Laboratory Animal Center (Shanghai, China). Animals were adjusted for a week. To eliminate Leydig cells from the testis, a rat received a single intraperitoneal injection of EDS (75 mg/kg of body weight), which was dissolved in a mixture of dimethyl sulfoxide (DMSO) solution: H_2_O (1: 3, v/v). For the experiment in vivo, Leydig cell‐depleted rats by EDS were divided into three groups. OSM was dissolved in normal saline for administration. Each testis of a rat received an intratesticular injection of 0, 10 or 100 ng/testis/day OSM (20 μL per testis) from post‐EDS day 14 for 14 days. To avoid the possible systemic effects of OSM on hypothalamus‐pituitary‐gonadal axis, intratesticular injection was adopted. Normal saline served as the control. This time‐course of administration was used because progenitor Leydig cells begin to emerge from SLCs on postnatal day 14 during regeneration.^9^, ^34^ Fourteen days after OSM treatment, rats were euthanized by CO_2_ and bloods were collected to obtain serum. The serum samples were stored at −20°C for testosterone, LH, and follicle‐stimulating hormone (FSH) measurement. One testis per rat was frozen in −80°C for qPCR, RNA‐seq sequencing, and Western blotting analysis. The contralateral testis was punched three holes by a 27G needle and fixed in Bouin's solution for 1 day for immunohistochemical and immunofluorescent stainings. All animal procedures were performed in accordance with the protocol approved by Animal Care and Use Committee of Wenzhou Medical University.

### 2.3 Serum testosterone assay in vivo

The objective of the assay is to investigate whether OSM affects the testosterone levels. The serum testosterone concentrations were measured by a chemiluminescence kit according to manufacturer's instruction (Siemens, Munich, Germany) as previously described.^35^ The minimal detection concentration was 0.2 ng/mL.

### 2.4 ELISA for serum LH and FSH levels in vivo

The objective of the assay is to quest the influence of serum LH and FSH levels after the treatment of OSM. Serum LH and FSH levels were detected with enzyme linked immunosorbent assay (ELISA) kit according to the manufacturer's instructions (Chemicon, Temecula, CA, USA) as described previously.^36^ Briefly, an aliquot of a sample and assay diluent were added to each well of the pre‐coated 96‐well plate. The plate was incubated for 2 hours at room temperature and washed 5 times with a washing buffer. Peroxidase‐conjugated IgG anti‐LH or anti‐FSH solution was added into each well for 2 hours at room temperature. Then, the plate was washed 5 times. Substrate buffer was added into each well and incubated in the dark place for 30 minutes at room temperature. The enzyme reaction was stopped by stop solution. The quantification of LH and FSH levels was obtained by a microplate reader at 550 nm with correction wavelength at 450 nm.

### 2.5 Preparation of RNA‐seq library in vivo

Total RNAs were extracted from testes using the Trizol Kit (Invitrogen, Carlsbad, CA, USA) according to the manufacturer's instruction. The concentration of total RNAs from each testis was measured using a NanoDrop ND‐1000 (ThermoFisher Scientific, Shanghai, China). An aliquot of 1‐2 μg total RNAs was used to prepare the sequencing library as follows: total RNAs were enriched by oligo‐dT magnetic beads and RNA‐seq library was prepared using KAPA Stranded RNA‐Seq Library Prep Kit (Illumina, San Diego, CA, USA), which incorporates dUTP into the second cDNA strand and renders the RNA‐seq library strand‐specific. The completed libraries were qualified with Agilent 2100 Bioanalyzer (ThermoFisher Scientific) and quantified by absolute quantification qPCR method. To sequence the libraries on the Illumina HiSeq 4000 instrument (Illumina), the barcoded libraries were mixed, denatured to single stranded DNA, captured on Illumina flow cell, amplified in situ, and subsequently sequenced for 150 cycles for both ends.

### 2.6 Sequencing in vivo

We performed sequencing of testis and biological pathway analysis (the following section) to address the pathway of OSM‐mediated actions in vivo. Image analysis and base calling were performed using Solexa pipeline v1.8 (Off‐Line Base Caller software, v1.8, Illumina, Foster City, CA, USA). Sequence quality was examined using the FastQC software.^37^ The trimmed reads (trimmed 5′, 3′‐adaptor bases) were aligned to reference genome using Hisat2 software.^38^ The transcript abundances for each sample was estimated with StringTie,^39^ and the FPKM^40^ value for gene and transcript levels were calculated with R package Ballgown.^41^ The differentially expressed genes and transcripts were filtered using R package Ballgown.^41^ The novel genes and transcripts were predicted from assembled results by comparing to the reference annotation using StringTie and Ballgown and the coding potential of those sequences was then assessed using CPAT.^40^ Alternative splicing events and plots were detected using rMATS.^42^ Principle Component Analysis and correlation analysis were based on gene expression level, Hierarchical Clustering, Gene Ontology, Pathway analysis, Gene Ontology, Pathway analysis, scatter plots and volcano plots were performed with the differentially expressed genes in R, Python or shell environment for statistical computing and graphics.

### 2.7 Biological pathway analysis

Biological pathway analysis was performed as previously described.^24^ The GenMAPP2.1 (San Francisco, CA, USA) was used to create a map of signal pathways for the potential pathways. We imported our statistical results into the program and illustrated biological pathways containing differentially expressed genes. The results of the differential gene expression profile were confirmed by qPCR.

### 2.8 Quantitative real‐time PCR (qPCR) in vivo and in vitro

We performed the qPCR of OSM‐treated samples to verify the sequencing data of the testes and to investigate the effects of OSM on the gene expression of the seminiferous tubules. The first strand of cDNA was synthesized and used as the template for qPCR as previously described.^43^ The mRNA levels of *Lhcgr*,* Scarb1*,* Star*,* Cyp11a1*,* Hsd3b1*,* Cyp17a1*,* Hsd17b3*,* Srd5a1*,* Hsd11b1*,* Dhh*,* Igf1*,* Pdgfa* and *Nr5a1* were analyzed using the SYBR Green qPCR Kit (Roche, Basel, Switzerland). The reaction mixture consisted of 7.5 μL SYBR Green Mix, 0.75 μL forward and 0.75 μL reverse primers, 0.02 μg diluted cDNAs, and 4 μL RNA‐free water. The procedure of qPCR was set as the follows: 95°C for 5 minutes, followed by 40 cycles of 95°C for 10 seconds, and 60°C for 30 seconds. The Bio‐Rad CFX Manager Software was used to analyze the qPCR data. The specificity of the fluorescence signal was determined by both melting curve analysis and gel electrophoresis. The mRNA levels were determined by a standard curve method. Ct values were collected for the standard curve and the target mRNA levels were calculated from the curve and were normalized to *Rps16*. The primers and gene names were listed in Table S1.

### 2.9 Western blotting analysis in vivo

We performed Western blotting analysis of OSM‐treated testes to investigate the effects of OSM on the protein expression of steroidogenesis‐related genes. Western blotting was carried out as previously described.^44^ Briefly, the testes were put into RIPA lysis buffer (Bocai Biotechnology, Shanghai, China) and homogenized. The protein concentrations of samples were measured using the BCA Protein Assay Kit (Takara, Japan) and bovine serum albumin as the protein standard. An aliquot (30 μg) of proteins was loaded and the proteins were electrophoresed on the 10% polyacrylamide gels containing sodium dodecyl sulfate and the proteins were transferred onto the nitrocellulose membrane. The membrane was blocked with 5% non‐fat milk in TBST buffer for 2 hours and incubated with primary antibodies against LHCGR, SCARB1, CYP11A1, HSD3B1, CYP17A1, HSD11B1, and ACTB at 4°C overnight. The membrane was washed and incubated with HRP‐conjugated anti‐rabbit (1:2000; Bioword, St. Louis Park, MN, USA) for 2 hours at room temperature. ACTB (the house‐keeping protein) served as a control. The protein levels were quantified using ImageJ software and normalized to ACTB. All the antibody information was listed in Table S2.

### 2.10 Immunohistochemical staining of the testis in vivo

We performed immunohistochemical staining of the testis and enumerated Leydig cells (the following section) to investigate the effects of OSM on Leydig cell number. One testis from each rat was used for immunohistochemical staining using Vectastain ABC kit (Vector Laboratories, Burlingame, CA, USA) according to the manufacturer's instructions. Five testes per group at each time point were used and testis samples were prepared and then embedded in paraffin as a tissue array. Tissue‐array samples were dehydrated in ethanol and xylene and then embedded in paraffin. Six micrometer‐thick transverse sections were mounted on glass slides. Antigen retrieval was performed by microwave irradiation for 10 minutes in 10 mmol L^−1^ (pH 6.0) of citrate buffer, after which endogenous peroxidase was blocked with 0.5% of H_2_O_2_ in methanol for 30 minutes. Sections were then incubated with CYP11A1 (a general biomarker of all Leydig cells) or 11β‐hydroxysteroid dehydrogenase 1 (HSD11B1, a specific biomarker for Leydig cells at the advanced stage)^34^ polyclonal antibody diluted 1:200 overnight. Diaminobenzidine was used for visualizing the antibody‐antigen complexes, positively labeling Leydig cells by brown cytoplasmic staining. Mayer hematoxylin was applied as counterstaining. The sections were then dehydrated in graded concentrations of alcohol and cover‐slipped with resin (ThermoFisher Scientific, Waltham, UK). Non‐immune rabbit IgG was used in the incubation of negative control sections with working dilution the same as the primary antibody. The cells with stainings of CYP11A1 or HSD11B1 were counted.

### 2.11 Enumeration of Leydig cell number in the testis in vivo

In order to enumerate CYP11A1‐positive or HSD11B1‐positive Leydig cell numbers, sampling of the testis was performed according to a fractionator technique as previously described.^45^ Each testis was cut in eight discs and two discs were randomly selected. Then discs were cut in four pieces and one piece was randomly selected from total eight pieces. These pieces of testis were embedded in paraffin in a tissue array as above. Paraffin blocks were sectioned in 6 μm‐thick sections. About ten sections were randomly sampled from each testis per rat. Sections were used for immunofluorescent staining. Using the live image of a digital camera, under a 10× objective, and starting at a fixed point of the “upper” sections, total microscopic fields per section were counted. The total number of Leydig cells was calculated by multiplying the number of Leydig cells counted in a known fraction of the testis by the inverse of the sampling probability.

### 2.12 Immunofluorescent staining of the testis in vivo

We performed immunofluorescent staining of the testis to investigate the effects of OSM on the proliferation of Leydig cells. As for immunofluorescent staining of the testis, sections were incubated with the primary antibody of proliferating cell nuclear antigen (PCNA, a biomarker of proliferating cells) for 60 minutes and then washed and incubated with the CYP11A1 antibody for double staining. Fluorescent secondary antibody (Alexa‐conjugated anti‐rabbit or anti‐mouse IgG, 1:500) were used after the primary antibody. Sections were counterstained with mounting medium containing DAPI. Sections were visualized under a fluorescent microscope (Olympus, Tokyo, Japan). The CYP11A1 (green color in the cytoplasm) was used to label Leydig cells and PCNA (red color in the nucleus) was used to label proliferating cells.

### 2.13 Isolation and culture of seminiferous tubules in vitro

We performed culture of seminiferous tubules (including the following two sections) and treated them to investigate the effects of OSM on the proliferation and differentiation of stem Leydig cells. One 70‐day‐old rat was injected intraperitoneally with a single dose of EDS (75 mg/kg body weight) for the in vitro experiment. The rat was killed by CO_2_ euthanization 7 days after EDS treatment, when all Leydig cells were eliminated.^6^, ^7^ Testes were placed in MEM‐199 medium and decapsulated. Seminiferous tubules were mechanically separated from the interstitium according to the described method.^15^ The tubules were distributed randomly into a 12‐well plate, with each well containing tubule fragments of equivalent total length (about 1 inch). Seminiferous tubules were cultured in Leydig cell differentiation medium (LDM),^13^ which contains DMEM/F12 medium (pH 7.2) supplemented with 0.1% BSA, 15 mmol L^−1^ HEPES, 2.2 mg/mL sodium bicarbonate and penicillin/streptomycin (100 U/mL and 100 μg/mL), insulin‐transferrin‐selenium (ITS), 5 ng/mL LH and 5 mmol/L lithium chloride, in a humidified atmosphere of 5% CO_2_ at 37°C in a 12‐well Costar culture plate with ultralow attachment surface (Corning, NY, USA) for up to 2 weeks. In LDM, different concentrations (0, 0.1, 1, 10, and 100 ng/mL) of OSM were added. We used S3I‐201 (5 mmol L^−1^) and filgotinib (10 nmol L^−1^), the antagonists of OSM, to explore the underlying mechanism of OSM. S3I‐201 is a STAT3 inhibitor,^46^ while filgotinib is a JAK1 inhibitor.^47^ After 2 weeks in LDM, the ability of the seminiferous tubules to produce testosterone was assayed by measuring testosterone concentration in the cultured medium as previously described.^48^

### 2.14 Stem Leydig cell proliferation assay in vitro

The proliferative capacity of stem Leydig cells was measured by the Click‐it EdU Alaxa Fluor Kit (Invitrogen, Carlsbad, CA, USA) according to the manufacturer's instruction as described.^23^ In brief, the seminiferous tubules were treated with OSM (0, 0.1, 1, 10, and 100 ng/mL) for a week. Then, an aliquot (2 μL) of 1:1000 diluted EdU was added to each well and incubated for 24 hours. Seminiferous tubules were washed twice with 500 μL PBS buffer containing 3% bovine serum albumin. The seminiferous tubules were then fixed in 500 μL 4% paraformaldehyde at room temperature for 30 minutes. Tubules were washed and incubated with reaction solution in the dark for 45 minutes. The sections were washed again and mounted on a slide for visualization under a fluorescent microscope (Olympus) and images were captured. EdU‐positive cells (green color) were counted and calculated by the percentage of EdU positive cells divided by the cells at the outer layer of the seminiferous tubule using the ImageProPlus 7.0 software (Media Cybernetics, Rockville, MD, USA).

### 2.15 Indirect SLC proliferative capacity assay in vitro

In order to perform the indirect proliferative assay of stem Leydig cells, the seminiferous tubules were cultured for 1 week to increase the number of stem Leydig cells and then the seminiferous tubules were switched into LDM for 2 weeks, where stem Leydig cells were induced to differentiate into adult Leydig cells to produce testosterone.^13^ If stem Leydig cell number increases or decreases after treatment of a niche factor, the testosterone levels produced by Leydig cells in the medium will reflect the proliferative capacity.^13^ Seminiferous tubules were treated with different concentration of OSM (0, 0.1, 1, 10, and 100 ng/mL) in M199 medium for a week, then the tubules were switched to LDM for 2 weeks and the media were collected and testosterone levels were assayed.

### 2.16 Progenitor Leydig cell culture and proliferation assay in vitro

To investigate the effect of OSM on the proliferative capacity of progenitor Leydig cells, we isolated progenitor Leydig cells and cultured them in the 12‐well plate with different concentrations of OSM (0, 1, 10, and 100 ng/mL) at the density of 5 × 10^5^ cells/well for 24 hours. The isolation of progenitor Leydig cells was performed as described previously.^49^ In brief, forty 21‐day‐old male Sprague Dawley rats were euthanized by CO_2_. Testes were taken and digested in 0.25 mg/mL collagenase D (Boehringer Mannheim Biochemicals, Indianapolis, IN, USA) at 34°C for 10 minutes and cell mixture was filtered through nylon mesh, followed by density gradient centrifugation in 55% isotonic Percoll at 25,000 × g for 45 minutes at 4°C. Progenitor Leydig cell fraction was picked between densities of 1.064 and 1.070 g/mL. Purity of progenitor Leydig cells fractions was judged by histochemical staining for HSD3B1 activity, with 0.4 μmol L^−1^ etiocholanolone as the steroid substrate as previously described.^50^ The purity of progenitor Leydig cells was typically over 95%. Progenitor Leydig cells were resuspended in phenol red‐free DMEM: F12 medium (Sigma, St. Louis, MO, USA) supplemented with 1 mg/mL BSA. Then, cells were incubated with Click‐it EdU Alaxa Fluor Kit for 2 hours. Cells were harvested and fixed with 4% paraformaldehyde for 15 minutes and permeabilized using Triton X‐100 solution for 30 minutes in the dark place at room temperature. Fixed cells were stained with the Click‐iT reaction mixture. Then, cell nucleus was stained with DAPI as contrast staining. Cells were observed under an inverted fluorescence microscopy.

### 2.17 Statistical analysis

Data were expressed as the mean ± SE, *P* < 0.05 was considered statistically significant. The differences of groups were evaluated by one‐way ANOVA followed by ad hoc Dunnett's multiple comparisons test to compare with the control using SigmaStat software (Systat Software Inc., Richmond, CA, USA).

## 3 RESULTS

### 3.1 OSM reduces testosterone levels in the Leydig cell regeneration model in vivo

**Figure 1:**
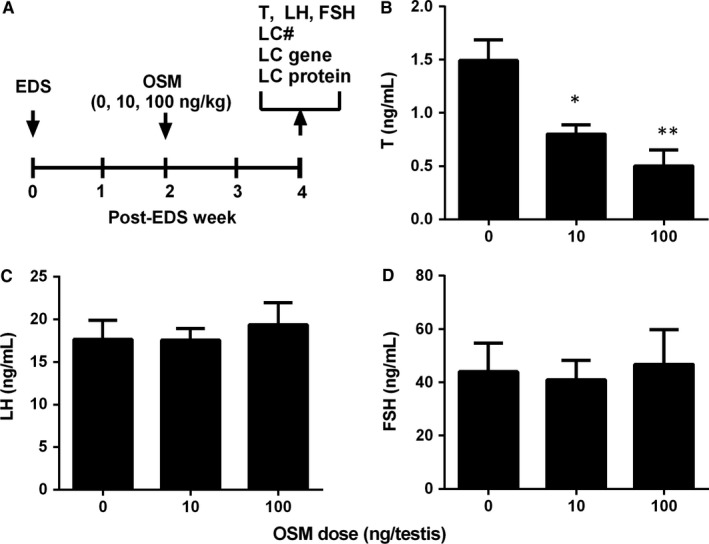
Oncostatin M (OSM) affects serum testosterone, LH, FSH levels in vivo. (A) Regimen of OSM treatment; (B) Serum testosterone (T); (C) LH; (D) FSH levels. Mean ± SE, n = 6; *P < 0.05, **P < 0.01 indicate significant differences in OSM group when compared to the control

We used a Leydig cell regeneration model to explore the effects of OSM on rat Leydig cell regeneration in vivo. A single intraperitoneal injection of 75 mg/kg EDS completely eliminates Leydig cells within 7 days,^51^, ^52^ with only stem Leydig cells being present in the interstitium of the testis.^9^ At post‐EDS day 21, progenitor Leydig cells are formed from the commitment of stem Leydig cells, and at post‐EDS day 28, progenitor Leydig cells differentiate into immature Leydig cells. We treated rats with EDS to eliminate Leydig cells, and intratesticularly injected OSM (0, 10, or 100 ng/testis/day) from post‐EDS day 14 to 28 (Figure 1A). After the treatment, OSM did not affect the body weights and testis weights when compared to the control (Table S3). OSM decreased serum testosterone levels at 10 and 100 ng/testis at post‐EDS day 28 (Figure 1B). However, it did not alter serum LH (Figure 1C) and FSH (Figure 1D) levels. These data suggest that OSM inhibits Leydig cell regeneration primarily via direct action on stem/progenitor Leydig cells.

### 3.2 Sequencing analysis reveals OSM‐mediated inhibition of Leydig cell regeneration in vivo

**Figure 2:**
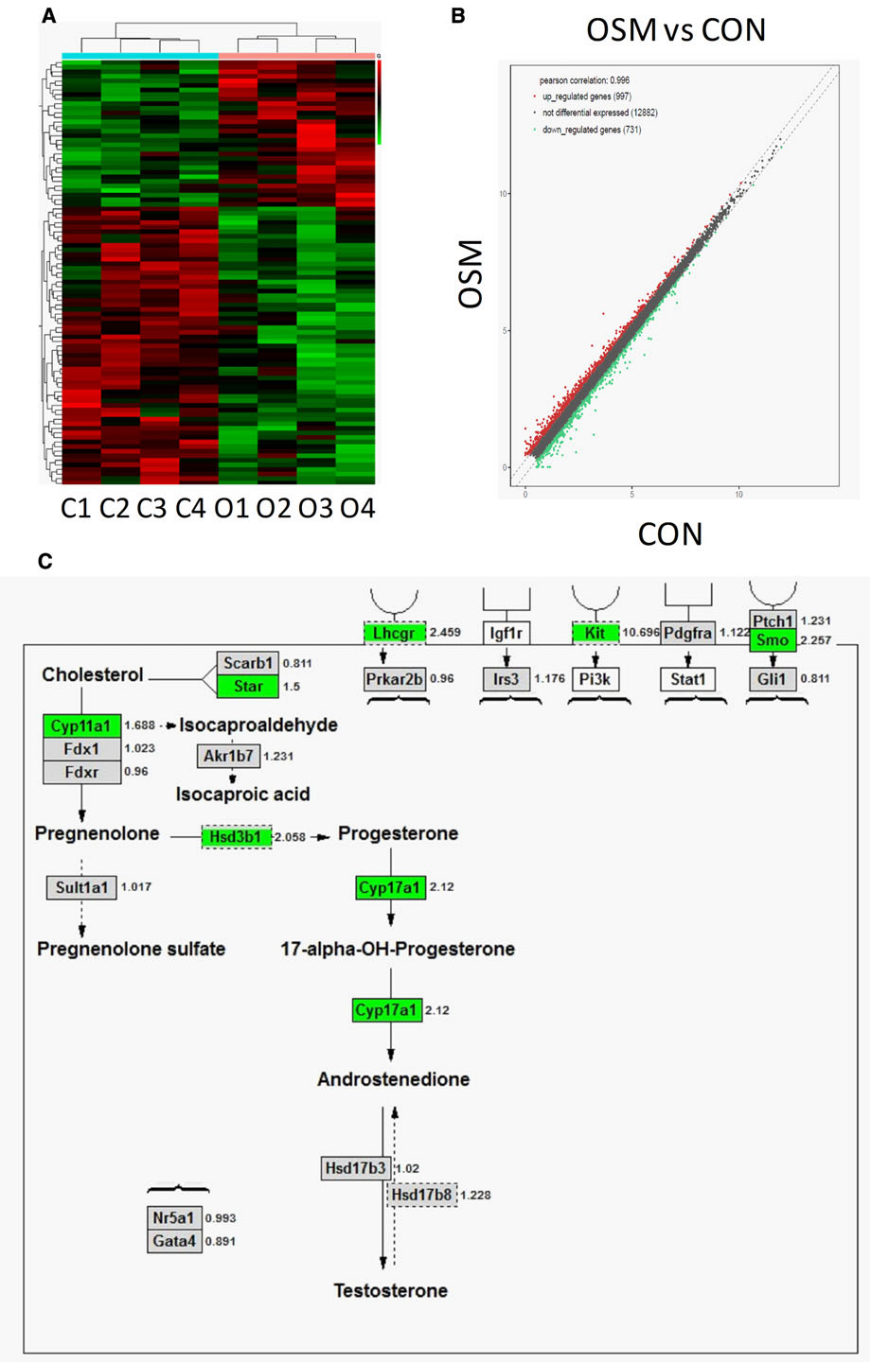
RNA‐seq analysis between oncostatin M (OSM) (O) and the control (C). (A) Heatmap of mRNAs between OSM (O, O1‐4) and control (C, C1‐4) samples; Red color = up‐regulated genes, Green color = down‐regulated genes; (B) Scatter analysis of mRNAs between OSM and control (CON) samples; (C) Steroidogenic pathway analysis, red color = up‐regulated genes at ≥1.5 folds, green color = down‐regulated genes at ≥1.5 folds; gray color = unchanged genes, while color = unmapped genes; digital number is the ratio of control over OSM, n = 4

We examined the effects of OSM (10 ng/testis) on Leydig cell gene expression levels using RNA sequencing analysis. We sequenced the transcripts and 17,745 transcripts were detected in the testis of two groups. Of these transcripts, 997 transcripts were significantly up‐regulated (*P* < 0.05) and 731 transcripts were significantly down‐regulated (*P* < 0.05) in the 10 ng/testis OSM group when compared to the control (Figure 2A and B). In the Leydig cell steroidogenic pathway, gene expression of several Leydig cell genes (*Star*,* Cyp11a1*,* Hsd3b1*, and *Cyp17a1*) was down‐regulated (Figure 2C). In the hormone or growth factor‐receptor complex, *Lhcgr*,* Kit*, and *Smo* were also significantly down‐regulated. This indicates that the Leydig cell regeneration is blocked.

### 3.3 OSM signaling pathway in vivo

We examined OSM signaling pathway via *Il6st*‐*Jak*‐*Stat3* pathway and found that the expression of the OSM downstream genes (*Shc1*,* Fyn*,* Prk2b*,* Inppl1*,* Gsk3b*, and *Pxn*) were significantly up‐regulated by over 1.5 folds (Figure S1). This indicates that OSM might activate OSM receptor‐IL6ST‐JAK‐STAT3 signaling pathway.

### 3.4 qPCR verification of OSM‐mediated down‐regulated Leydig cell gene expression in vivo

**Figure 3:**
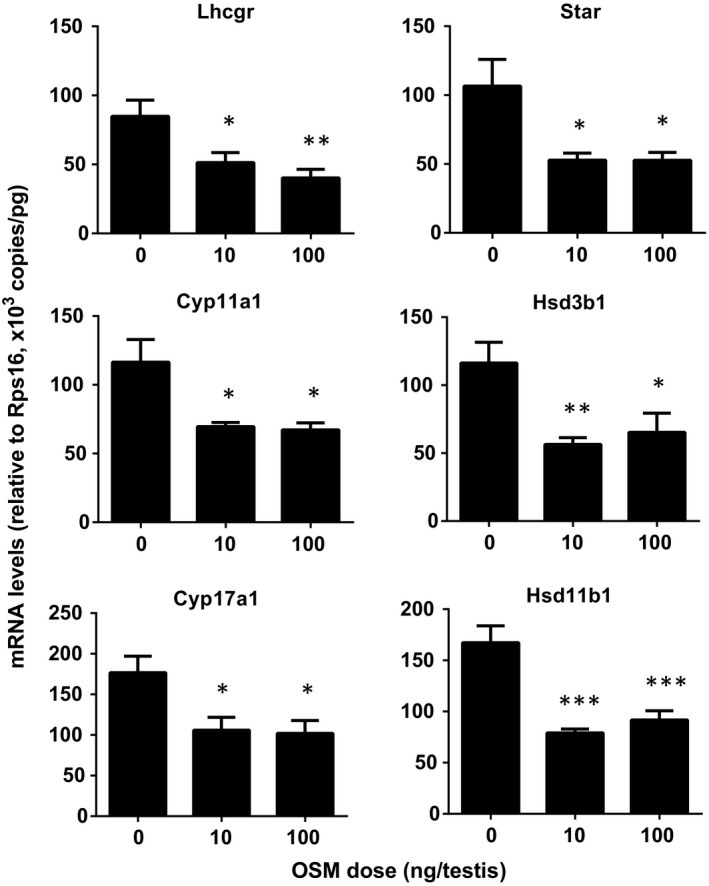
Oncostatin M (OSM) down‐regulates the expression levels of Leydig cell‐specific genes in vivo. The mRNA levels of *Lhcgr*,* Star*,* Cyp11a1*,* Hsd3b1*,* Cyp17a1* and *Hsd11b1* were analyzed by qPCR in testes from the rats treated with 0, 10 and 100 ng/testis OSM on post‐EDS day 14 for 28 days. Mean ± SE, n = 6, *P < 0.05, **P < 0.01 and ***P < 0.001 indicate significant differences when compared to the control

We found that OSM significantly reduced *Lhcgr, Star*,* Cyp11a1, Hsd3b1, Cyp17a1*, and *Hsd11b1* levels at doses of ≥10 ng/testis OSM (Figure 3). OSM did not affect *Scarb1, Hsd17b3, Srd5a1, Dhh, Igf1, Pdgfa*, and *Nr5a1* mRNA levels (data not shown). This suggests that OSM selectively down‐regulates some Leydig cell gene expression.

### 3.5 OSM decreases Leydig cell protein levels during regeneration in vivo

**Figure 4:**
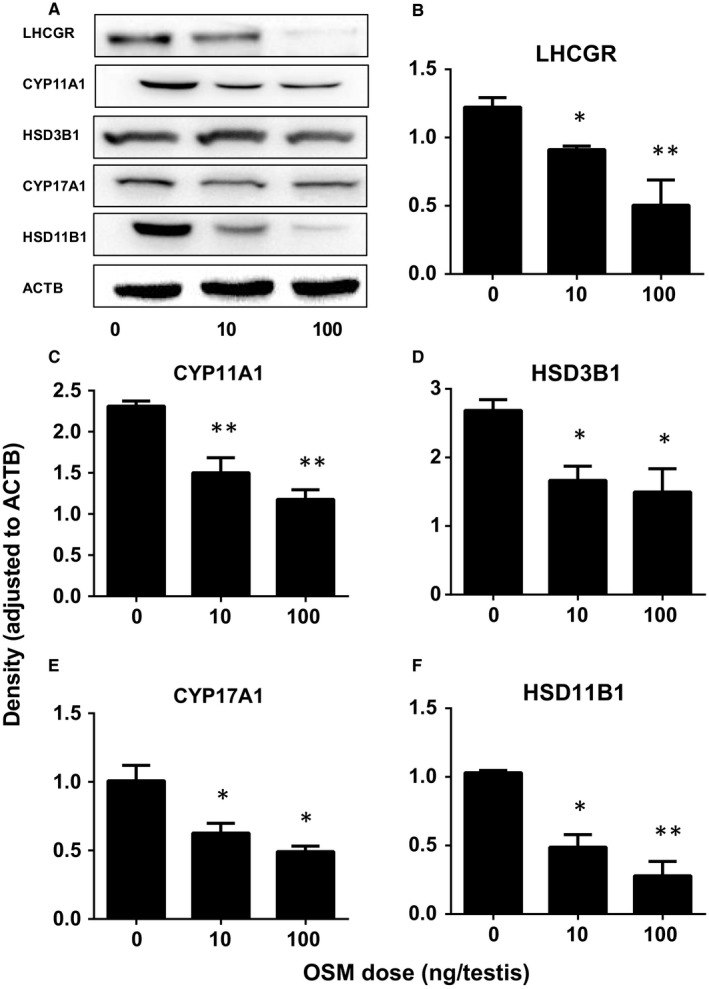
Oncostatin M (OSM) affects Leydig cell‐specific protein levels in vivo. (A) gel; (B‐F): quantitative data. The protein levels of LHCGR, CYP11A1, HSD3B1, CYP17A1, HSD11B1 and ACTB (internal control) were analyzed by Western blot in testes from the rats treated with 0, 10, and 100 ng/testis OSM on post‐EDS day 14 for 28 days. Mean ± SE, n = 3, *P < 0.05, **P < 0.01 indicate significant differences when compared to the control

We also detected the protein levels of Leydig cell gene products by Western blot and the result showed that LHCGR, CYP11A1, HSD3B1, CYP17A1 and HSD11B1 levels were lower in OSM‐treated testis, which was similar to the respective mRNA levels (Figure 4).

### 3.6 OSM does not affect the proliferation of Leydig cells in vivo

We used two biomarkers to label the Leydig cells: CYP11A1 (representing all the Leydig cells) and HSD11B1 (representing Leydig cells at the advanced stage).^11^, ^12^ We found that OSM did not affect both CYP11A1‐positive and HSD11B1‐positive cell numbers at ≥10 ng/testis (Figure S2). This indicates that OSM might not affect Leydig cell proliferation. Indeed, we further detected the PCNA‐positive Leydig cells via PCNA‐CYP11A1 double staining and found that OSM did not increase PCNA‐positive Leydig cell number (Figure S3). This confirms that OSM does not affect Leydig cell proliferation.

### 3.7 OSM blocks stem Leydig cell differentiation in vitro

**Figure 5:**
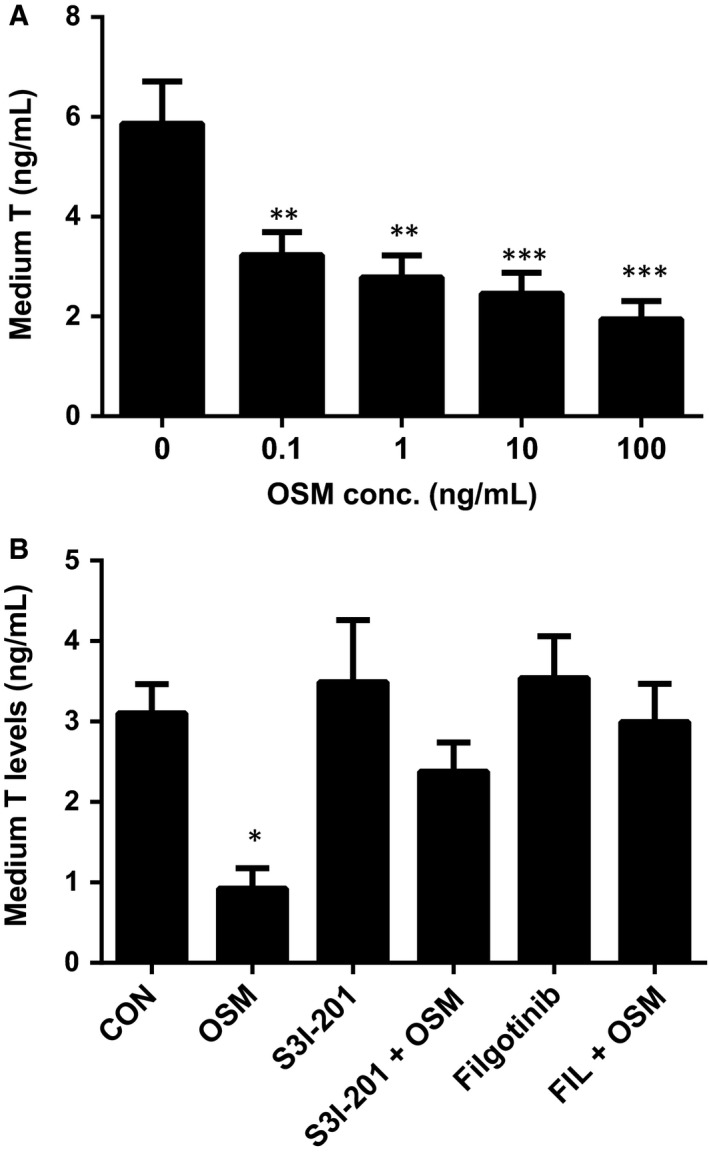
Effects of oncostatin M (OSM) on medium testosterone levels after in vitro stem Leydig cell differentiation. The seminiferous tubules were cultured in LDM medium, being induced to differentiate into Leydig cells to secrete testosterone. A, concentration‐dependent treatment of OSM (0, 0.1, 1, 10 and 100 ng/mL); B, treatment of OSM (10 ng/mL) with or without S3I‐201 (a STAT3 antagonist, 5 μmol L^−1^) or filgotinib (a JAK1 inhibitor, 10 nmol L^−1^). Mean ± SE, n = 6. *, **, *** indicate significant differences between two groups when compared to the control at *P* < 0.05, 0.01, 0.001, respectively

Since the Leydig cell regeneration during the 28‐day period after EDS treatment covers the differentiation of stem into progenitor and immature Leydig cells,^11^ we ask whether OSM blocks the differentiation of stem Leydig cells into the Leydig cell lineage. We previously developed an in vitro stem Leydig cell development model using the seminiferous tubule culture.^13^ As shown in Figure 5A, OSM concentration‐dependently inhibited steroidogenesis at doses of ≥0.1 ng/mL. In order to test the underlying mechanism, we treated stem Leydig cells with S3I‐201 (a STAT3 antagonist) or filgotinib (a JAK1 inhibitor) alone or in combination with 10 ng/mL OSM (Figure 5B). It showed that S3I‐201 and filgotinib alone did not affect the medium testosterone levels. However, both S3I‐201 and filgotinib reversed OSM‐mediated suppression of steroidogenesis (Figure 5B). These data indicate that OSM blocks stem/progenitor Leydig cell differentiation in vitro via JAK1‐STAT3 pathway.

### 3.8 OSM blocks stem Leydig differentiation by down‐regulating Leydig cell gene expression in vitro

**Figure 6:**
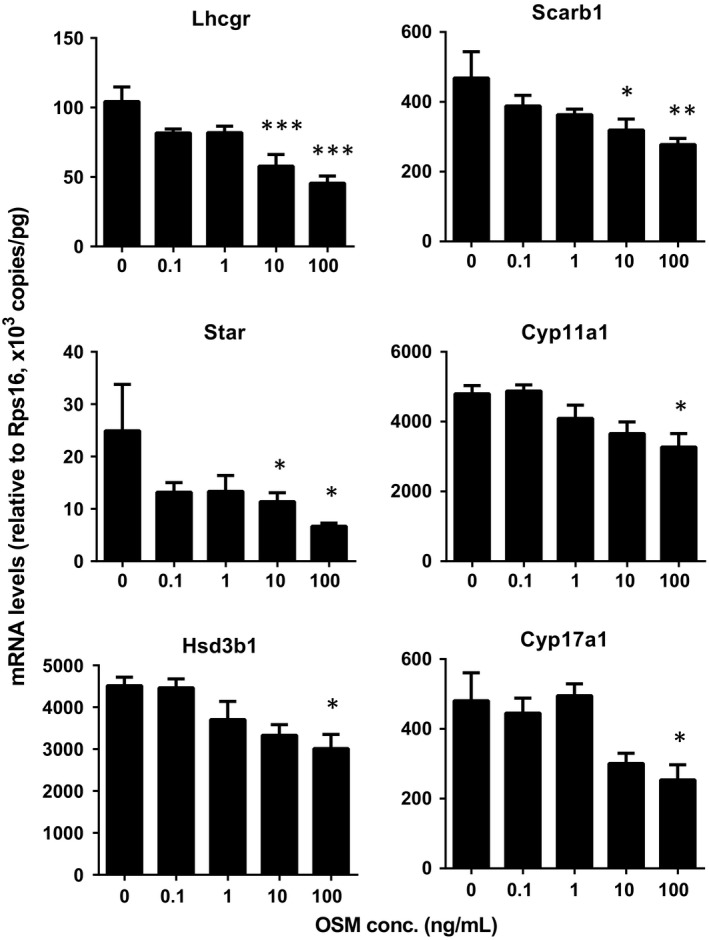
Oncostatin M (OSM) downregulates Leydig cell gene expression in vitro. Seminiferous tubules were treated with OSM (0, 0.1, 1, 10 *and* 100 nmol/L) for 1 week. The mRNA levels of *Lhcgr*,* Scarb1*,* Star*,* Cyp11a1*,* Hsd3b1* and *Cyp17a1*. *Rsp16* served as the internal control. Mean ± SE, n = 6‐12, *P < 0.05, **P < 0.01, ***P < 0.001 indicate significant differences when compared to the control

We measured the mRNA levels of *Lhcgr*,* Scarb1*,* Star*,* Cyp11a1*,* Hsd3b1, Cyp17a1, Hsd17b3*,* Srd5a1* and *Hsd11b1*. We found that at ≥10 ng/mL OSM down‐regulated *Lhcgr*,* Scarb1* and *Star* levels and at 100 ng/mL it also down‐regulated *Cyp11a1*,* Hsd3b1* and *Cyp17a1* levels (Figure 6). This indicates that OSM inhibits stem Leydig cell differentiation by down‐regulating Leydig cell specific gene expression.

### 3.9 OSM does not alter stem Leydig cell proliferation in vitro

We used EdU incorporation into the proliferating cells to label the dividing stem Leydig cells on the surface of the seminiferous tubules. As shown in Figure S4, OSM did not alter the number of EdU‐positive SLCs, indicating that OSM did not affect the proliferation of stem Leydig cells. In the presence of LDM, the stem Leydig cells can be induced into adult Leydig cells, producing testosterone. After 1 week of OSM treatment in M199 medium and then the seminiferous tubules were switched to the LDM and cultured for 2 weeks to induce the differentiation of the stem Leydig cells into adult Leydig cells that secrete testosterone in the medium. OSM did not change the medium testosterone level after the Leydig cell differentiation induction (Figure S4), which confirms that OSM does not affect stem Leydig cell proliferation. In addition, we also used EdU incorporation to label the progenitor Leydig cells in vitro. As shown in Figure S5, OSM did not alter the number of EdU‐positive progenitor Leydig cells, indicating that OSM did not affect the proliferation of progenitor Leydig cells.

## 4 DISCUSSION

EDS, a rat Leydig cell eliminator,^53^, ^54^, ^55^ can deplete all Leydig cells, decreasing intratesticular testosterone to the undetectable level and can induce a wave of Leydig cell regeneration in the adult rat testis.^56^, ^57^ On post‐EDS day 14,^58^ there are no Leydig cells in the rat testis. Progenitor Leydig cells appear from the differentiation of stem Leydig cells on post‐EDS day 21 and 7 days later immature Leydig cells emerge from the maturation of progenitor Leydig cells.^11^ On post‐EDS day 28, Leydig cells show the typical characteristics of immature Leydig cells, which have the expression of the Leydig cell specific genes (*Lhcgr, Scarb1, Star, Cyp11a1, Hsd3b1, Cyp17a1, Hsd17b3, Hsd11b1* and *Insl3*).^11^, ^58^ The present results showed that OSM inhibited the regeneration of Leydig cells in vivo and the differentiation of stem Leydig cells in vitro, with the evidence of the reduced serum testosterone levels and lower expression levels of *Lhcgr, Star, Cyp11a1, Cyp17a1, Hsd3b1* and *Hsd11b1* and their proteins in vivo and the down‐regulation of *Lhcgr, Star, Cyp11a1, Cyp17a1, Hsd3b1* and *Cyp17a1* expression in vitro. OSM did not affect the proliferation of Leydig cells, because it did not change the Leydig cell number and PCNA‐labeling index and did not affect EdU incorporation into primary progenitor Leydig cells in vitro and stem Leydig cells on the surface of the seminiferous tubules. OSM exerts its action via JAK1‐STAT3 pathway, as evidence that JAK1 and STAT3 antagonists reversed OSM action in vitro.

Leydig cells have been demonstrated to secrete IL‐6^59^ and to possess IL‐6 and LIF receptors,^60^, ^61^ indicating that some members of this family of cytokines may be involved in the proliferation and/or differentiation of this cell type. In the present study, we also demonstrated that OSM could affect differentiation of Leydig cells in vivo and in vitro. Our in vitro study clearly demonstrated that OSM exerted its suppression of stem Leydig cell differentiation via JAK1‐STAT3 signal pathway after using STAT3 and JAK1 antagonists (Figure 5). Using RNA‐seq analysis in the OSM‐treated testis, we also demonstrated that some mRNAs in IL6ST‐JAK1‐STAT3 signal pathway were significantly up‐regulated (Figure S1). OSM binds to its receptor complex, the type I or type II OSM receptor, and activates the JAKs and the activated JAKs in turn activates downstream pathway STAT3.^62^ It has been demonstrated that the type I OSM receptor is mainly present in stem and progenitor Leydig cells in the rat testis.^30^ This indicates that stem and progenitor Leydig cells are the target cells of OSM. However, it is still unknown how JAK1‐STAT3 pathway results in OSM‐mediated blockade of stem/progenitor Leydig cell differentiation. NR5A1 (encoded by *Nr5a1*) is a major transcription factor to regulate the commitment of stem Leydig cells into the Leydig cell lineage.^63^ However, in the present study, we did not find that the expression of *Nr5a1* was altered after the treatment of OSM (Figure 2C), indicating that the effects of OSM on stem Leydig cell development may exert via other mechanisms.

Sertoli cells secrete many niche factors to regulate the Leydig cell development.^4^ Therefore, we examined whether OSM affected the expressions of *Dhh*,* Pdgfa* and *Igf1* in the Sertoli cells. We did not find any changes of the expression levels of these genes after the treatment of OSM, which suggests the effects of OSM on stem Leydig cell development via a direct mechanism. After RNA‐seq analysis, we found that OSM mainly down‐regulated the expression of *Lhcgr*,* Kit* and *Smo* (Figure 2C). LHCGR (encoded by *Lhcgr*) is the receptor of LH, which is critical for the regeneration of Leydig cells after EDS treatment and differentiation of SLCs.^64^, ^65^ In *Lhcgr*‐null mice, Leydig cells stay in the progenitor stage without differentiation into the adult Leydig cells.^65^, ^66^ In the EDS‐treated Leydig cell regeneration model, when the blockade of LH levels using testosterone implant prevents the regeneration of Leydig cells on post‐EDS day 28 and administration of human chorionic gonadotropin (hCG), a homolog of LH, stimulates the formation of immature Leydig cells.^67^ In the in vitro seminiferous tubule culture system, Leydig cells cannot be formed in LH‐free medium and supplement of LH to the medium significantly stimulates the differentiation of progenitor into adult Leydig cells, which are able to produce testosterone robustly.^22^ This indicates that LHCGR is critical for the differentiation of Leydig cells once progenitor Leydig cells are committed. *Kit* encodes a receptor for Kit ligand, which was found to stimulate Leydig cell maturation.^22^, ^68^ Low concentration (1 ng/mL) of Kit ligand significantly induces the differentiation of stem into progenitor Leydig cells via increasing the expression level of steroidogenic acute regulatory protein (Star).^22^ DHH signaling is very critical for the differentiation of Leydig cells after it binds to PITCH1 (encoded by *Pitch1*) to activate SMO to stimulate Leydig cell differentiation. Knockout of *Dhh* in mice blocks Leydig cell differentiation during puberty.^19^ Treatment of the Leydig cell‐free seminiferous tubules or CD90‐positive stem Leydig cells by a SMO agonist significantly stimulates the differentiation of stem into the Leydig cell lineage.^13^ Therefore, the significant down‐regulation of these genes (*Lhcgr*,* Kit* and *Smo*) by OSM contributes to the blockade of Leydig cell development.

Interestingly, in the rat testis, OSM has been reported to be present in the late Leydig cell lineage.^30^ OSM itself stimulates stem and progenitor Leydig cell differentiation but blocks the LH‐mediated stimulation.^30^ In this regard, Leydig cells in the advanced stages secrete OSM, which negatively controls the maturation of stem/progenitor Leydig cells to counterbalance of population of Leydig cells in the testis once LHCGR is formed in the progenitor Leydig cells.

**Figure 7:**
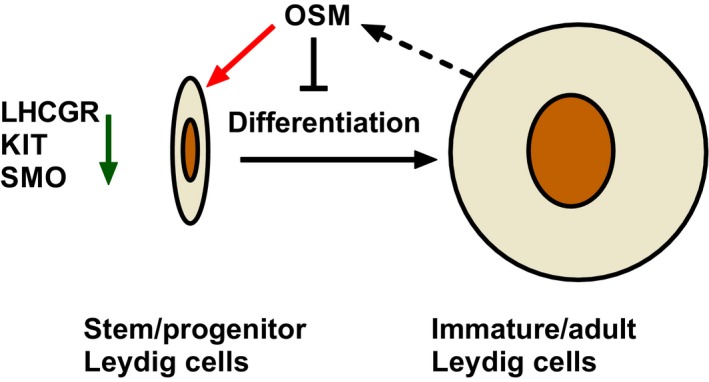
Illustration of the working mechanisms for oncostatin M (OSM) in regulation of Leydig cell differentiation. Immature/adult Leydig cells secrete OSM, which binds to the OSM type I receptor on stem/progenitor Leydig cells, activating IL6ST‐JAK1‐STAT3 signaling, leading to the down‐regulation of LHCGR, KIT and SMO that are critical for Leydig cell differentiation. Black dash arrow, secretion; Red arrow, binding to the receptor; Black “T”, inhibition; Green arrow, down‐regulation

In summary, we demonstrate for the first time that OSM secreted by adult Leydig cells blocks stem or progenitor Leydig cell differentiation via IL6ST‐JAK1‐STAT3 signaling pathway, which leads to down‐regulation of LHCGR, KIT and SMO that are critical factor for Leydig cell development (the schema is illustrated in Figure 7).

## COMPETING INTERESTS

The authors declared that no competing interests exist.
