# Supplementary material for: Oncostatin M inhibits differentiation of rat stem Leydig cells in vivo and in vitro
Source: J Cell Mol Med. 2018 Oct 15;23(1):426–38. doi: 10.1111/jcmm.13946 (PMC6307848; doi:10.1111/jcmm.13946)
Supplement: Supplementary file 7 [file JCMM-23-426-s007.docx]

**Supplementary Table S2 Antibody information**

| **Antibody** | **Species** | **Vendor (City, State)** | **Dilution** | |
| --- | --- | --- | --- | --- |
|  |  |  | **WB** | **HS** |
| Actin (ACTB) | Rabbit | Cell Signaling Technology (Danvers, MA) | 1:1000 | ND |
| LHCGR | Rabbit | Multi Sciences (Hangzhou, China) | 1:1000 | ND |
| HSD3B1 | Rabbit | Multi Sciences (Hangzhou, China) | 1:500 | ND |
| CYP17A1 | Rabbit | Abcam (San Francisco, CA) | 1:1000 | ND |
| CYP11A1 | Rabbit | Cell Signaling Technology (Danvers, MA) | ND | 1::200 |
| HSD11B1 | Rabbit | Abcam (San Francisco, CA) | ND | 1:200 |
| BCL-2 | Rabbit | Cell Signaling Technology (Danvers, MA) | 1:1000 | ND |
| BAX | Rabbit | Absin (Shanghai, China) | 1:1000 | ND |

WB = Western blot; HS = Histochemical staining; ND = Not detected.
