# Supplementary material for: Oncostatin M inhibits differentiation of rat stem Leydig cells in vivo and in vitro
Source: J Cell Mol Med. 2018 Oct 15;23(1):426–38. doi: 10.1111/jcmm.13946 (PMC6307848; doi:10.1111/jcmm.13946)
Supplement: Supplementary file 8 [file JCMM-23-426-s008.docx]

**Supplementary Table S3. Body weight and testis weight**

| Parameters | | **Dosage (ng/testis)** | | |
| --- | --- | --- | --- | --- |
|  |  | **0** | **10** | **100** |
| **Body weight (g)** | | 380. 7±11.76 | 370.2±8.408 | 375.8±13.83 |
| **Testis weight (g)** | | 1.345±0.09173 | 1.275±0.1068 | 1.122±0.1014 |

Mean ± SEM, n=6-12.
